# Changes in the growth performance, serum biochemistry, rumen fermentation, rumen microbiota community, and intestinal development in weaned goats during rumen-protected methionine treatment

**DOI:** 10.3389/fvets.2024.1482235

**Published:** 2024-11-08

**Authors:** Youli Wang, Wei Liu, An Li, Wuqie Qubi, Chengsi Gong, Xuening Li, Jiani Xing, Haitao Shi, Yanyan Li, Yong Wang, Yaqiu Lin

**Affiliations:** ^1^College of Animal Science and Veterinary, Southwest Minzu University, Chengdu, China; ^2^Key Laboratory of Qinghai-Tibetan Plateau Animal Genetic Resource Reservation and Utilization, Ministry of Education, Southwest Minzu University, Chengdu, China; ^3^Key Laboratory of Sichuan Province for Qinghai-Tibetan Plateau Animal Genetic Resource Reservation and Exploitation, Southwest Minzu University, Chengdu, China

**Keywords:** goat, rumen-protected methionine, growth performance, rumen microbiota, serum biochemistry, small intestine

## Abstract

Rumen-protected methionine (RPM) such as coated methionine (CM) and 2-hydroxy-4-(methylthio)-butanoic acid isopropyl ester (HMBi) was usually used in dairy cows, but how RPM affects meat goats remains unclear. In this study, thirty weaned male Jianzhou Da’er goats were randomly assigned to one of three treatments: fed basal diet or basal diet supplemented with 0.12% CM or 0.22% HMBi, with the aim of examining their impact on growth performance, serum biochemistry, rumen fermentation, rumen microbiota, and intestinal development in meat goats. The findings indicate that HMBi supplementation led to an increase in body weight, feed intake, and feed-to-gain ratio, whereas CM only resulted in an increase in feed intake (all *p* < 0.05). Both CM and HMBi resulted in an increase in serum total cholesterol (TC), blood urea nitrogen (BUN), alkaline phosphatase (ALP), and aspartate aminotransferase (AST), albeit with a decrease in serum triglycerides (TG) and *β*-hydroxybutyric acid (BHB, all *p* < 0.05). Both CM and HMBi supplementation decreased the rumen butyric acid concentration (both *p* < 0.05). The 16S rRNA sequencing showed that HMBi supplementation significantly increased the total abundance of *Bacteroidetes* and *Firmicutes*. Both CM and HMBi supplements increased the abundance of *Rikenella* and *Proteiniphilum* but decreased the abundance of *Eisenbergiella*, *Enterocloster*, *Massilioclostridium*, *Eubacterium*, *Angelakisella*, *Blastopirellula*, *Christensenella,* and *Pseudoruminococcus*. CM supplementation specifically increased the abundance of *Desulfobulbus*, *Sodaliphilus,* and *Coprococcus* while decreasing the prevalence of *Anaerocella*, *Mogibacterium,* and *Collinsella*. The supplementation of HMBi significantly enhanced the abundance of *Paraprevotella*, *Bacilliculturomica*, *Lachnoclostridium*, *Dysosmobacter*, *Barnesiella*, and *Paludibacter*, while decreasing the abundance of *Butyrivibrio* and *Pirellula*. Moreover, the administration of both CM and HMBi supplementation resulted in an increase in the ammonia-producing and sulfate-reducing bacteria, whereas a decrease was observed in the ammonia-oxidating, health-associated, and disease-associated bacteria. Correlational analysis revealed that TG and BHB had a positive correlation with disease-associated and ammonia-oxidating bacteria, whereas they had a negative correlation with ammonia-producing bacteria. The serum BUN, ALP, and AST were positively correlated with ammonia-producing bacteria but were negatively correlated with ammonia-oxidating bacteria. Furthermore, both CM and HMBi supplementation improve the development of the small intestine, with HMBi having a better effect. In summary, this study indicates that both CM and HMBi supplementation improve lipid metabolism, nitrogen utilization, and intestinal development. The growth promotion effect of HMBi supplementation may be attributed to the increased abundance of volatile fatty acid-producing and nitrogen-utilizing bacteria and improved intestinal development.

## 1 Introduction

Methionine is a limited amino acid for ruminant that can be degraded by rumen microbes. In order to avoid the degradation of the rumen microbe, rumen-protected methionine (RPM) has been well developed, such as coated methionine (CM) and 2-hydroxy-4-(methylthio)-butanoic acid isopropyl ester (HMBi). CM can largely avoid degrading by the rumen microbe and then being absorbed by the hindgut. The ingested HMBi can be absorbed by the rumen wall and hindgut or degraded by the rumen microbe. HMBi can be transported as blood to almost all body tissues and organs, such as the rumen, omasum, kidney, and liver, and then converted to methionine and acetone (1, 2). Although RPM has a higher efficiency in providing methionine (2), a portion of them still undergo degradation in the rumen, a crucial digestive organ in ruminants. This may influence the rumen fermentation and rumen microbes. Furthermore, the small intestine is an important site for absorbing microbial protein that is produced in the rumen (3). A study has shown that methionine benefits intestinal organoid development (4). However, the effects of RPM supplementation on rumen fermentation, rumen microbial populations, and intestinal development remain unclear.

Currently, RPM has been extensively studied in dairy cattle and has been shown to have a positive impact on milk fat and protein (5). However, there have been few studies carried out on meat ruminants. In cattle, Qin et al. (6) reported that a diet supplemented with HMBi at approximately 0.3% increased the body weight in fatting cattle. However, CM supplementation had no impact on the growth performance in Fleckvieh bulls feeding CM (7). So far, the feeding period of studies of RPM in goats and sheep has varied from 30 to 80 days, the level of HMBi has varied from 0.05 to 0.2% DM, and the level of CM has been 0.1 ~ 1.15% (8–15). However, there was no improvement observed in goats. The feeding period may have an impact on the efficacy of RPM. Therefore, it is of greater significance to investigate the alterations in rumen fermentation, rumen microbe, serum biochemistry, and intestinal development when improved growth performance appears during feeding RPM.

The Jianzhou Da’er goat is a new breed formed by crossing Nubian goats with native goats from Jianyang City, Sichuan Province, in China. The meat is known for its tender texture and light odor, which has gained increasing attention from consumers. However, there is limited information about the Jianzhou Da’er goats. The objective of this study was to investigate the effects of different sources of RPM on growth performance, serum biochemistry, rumen fermentation parameters, rumen microbes, and intestinal development of weaned Jianzhou Da’er goats when improved growth performance appeared.

## 2 Materials and methods

This study was conducted at the Sichuan Tiandi Goat Biological Engineering Co., Ltd. (Chengdu, China). The ethical approval for the animal trial in this study was granted by the Animal Care and Ethics Committee of Southwest Minzu University (Approval code: SMU-202401047).

### 2.1 Experimental design and diet

A total of thirty weaned male Jianzhou Da’er goats (about 2.5 months old) with similar body weight (15.7 ± 0.19 kg) were randomly assigned to one of three treatments: fed basic diet (CT), dietary dry matter (DM) with 0.12% CM (available DL-methionine ≥85%; Mepron, Evonik Industries, Frankfort, Germany), or dietary DM with 0.22% HMBi (available content ≥46%; Metasmart, Adisseo, Paris, France). Each treatment contained 10 goats, each of which was individually housed and provided with free access to fresh water. The level of supplementation for coated methionine is based on the findings of a previous study (16). The inclusion of CM and HMBi was determined to provide an additional 0.1% metabolizable methionine in dietary DM. The diet was formulated according to the recommendations of the National Research Council (17), and the formulation and nutrients are shown in Table 1. Feeding was provided three times per day (08:00, 14:00, and 18:00). The concentration feed was provided at 18:00 with a supplemented RPM. The experiment lasted for 4.5 months, with the first 7 days used for preadaptation. The body weight was recorded on a monthly basis. Feed intake was recorded every day.

**Table 1:** Ingredients and nutrient composition of the diet (DM basis).

Feed ingredient	Composition (%)
Corn	20.09
Soybean meal	5.68
Rapeseed meal	4.86
Peanut vine	40.30
Corn stalk silage	26.87
Premix^a^	1.48
NaHCO_3_	0.72
Nutrients (%, DM)	
Organic matter	84.60
Crude protein	12.94
Metabolizable energy^b^ (Mcal/kg)	2.29
Ether extract	2.87
Neutral detergent fiber	31.93
Acid detergent fiber	25.72
Calcium	0.30
Phosphorus	0.25

a Premix composition per kg diet: 140000 ~ 500,000 IU vitamin A, 49000 ~ 200,000 IU vitamin D3, ≥ 300 mg Nicotinic acid, ≥ 1 mg biotin, ≥ 400 IU vitamin E, 0.8 ~ 7.5 g Fe as FeSO_4_·H_2_O, 0.4 ~ 6.2 g Mn as MnSO_4_, 160 ~ 370 mg Cu as CuSO_4_·5H_2_O, 800 ~ 6,000 mg Zn as ZnSO_4_·H_2_O, 8 ~ 25 mg Se as NaSeO_3_, 6 ~ 125 mg Co as CoCl2·6H_2_O, 8 ~ 125 mg I as KIO_3_, 80 ~ 200 g Ca as CaCO_3_, 0.3 ~ 6 g P as CaHPO_4_·2H_2_O, 60 g NaCl.

b Calculated from the methodology (78).

### 2.2 Sampling systems

After feeding for 4.5 months, blood samples were collected using a vacuum blood collection tube after fasting for 12 h. In each treatment, the rumen fluid from 10 goats was filtered by 4 layers of sterilized gauze. A portion of the rumen fluid was frozen using liquid nitrogen and stored at −80°C for the purpose of determining the VFA and rumen microbiota. A portion of the rumen fluid was used to determine the pH.

### 2.3 Serum biochemistry analysis

The blood was placed at room temperature for a duration of 4 h, followed by centrifugation at 3000 *g* for 10 min at 4°C. Serum total cholesterol (TC), triglycerides (TG), blood urea nitrogen (BUN), *β*-hydroxybutyric acid (BHB), total protein (TP), albumin (ALB), globulin (GLB), alkaline phosphatase (ALP), alanine transaminase (ALT), aspartate aminotransferase (AST), and lactic dehydrogenase (LDH) were detected according to the instructions provided by the commercial kits (Mindray, Shenzhen, China).

### 2.4 Rumen fermentation parameters

The freshly collected rumen fluid was examined for pH using a portable pH meter (Testo 206 pH2, Schwarzwald, Germany). Remen fluid stored at −80°C was used to analyze the concentration of VFA using a gas chromatograph (Agilent Technologies 7890B, California, America). The column used was an HP-INNOWax capillary column (30 m length, 0.32 mm diameter, and 0.5 μm film thickness; Agilent, California, America). The temperature of the column oven was 120°C, the equilibration time was 3 min, and then increased 10°C every 10 min to stabilize at 180°C. The temperature of the front injection port was 220°C, and the front detector was 250°C. The internal standard was 2-ethylbutanoic acid. Each rumen fluid sample was examined twice.

### 2.5 16S rRNA sequencing

For each treatment, 10 samples in each treatment were used for 16S rRNA sequencing. As described by He et al. (17, 18), microbial DNA from rumen fluid samples was extracted by an E.Z.N.A.^®^ DNA Kit (Norcross, Georgia, America) following the manufacturer’s instructions. The bacterial 16S ribosomal RNA genes were amplified through polymerase chain reaction (PCR). All amplicon sequencings were performed by Shanghai Biozeron Biotechnology Co., Ltd. (Shanghai, China). PacBio raw reads were processed using the SMRT Link Analysis software version 9.0 to obtain demultiplexed circular consensus sequence reads with the following settings: minimum number of passes = 3, minimum predicted accuracy = 0.99. Operational taxonomic units (OTUs) were clustered with a 98.65% similarity cutoff using UPARSE (version 7.1), and chimeric sequences were identified and removed using UCHIME.

### 2.6 Hematoxylin–eosin staining

After 48 h of fixing with 4% paraformaldehyde, the ileums and jejunums were dehydrated with increasing concentrations of ethanol (75, 85, 95, and 100%), cleared with xylene, and embedded in paraffin. Paraffin sections of the small intestine (5 μm) were then stained with the hematoxylin and eosin kits. Villus height (VH) and crypt depth (CD) were determined under a microscope (Olympus, Tokyo, Japan), and the typical field of view was photographed. Each slice was examined for approximately 10 villi and crypts, and the average VH and CD of each slice was used for statistical analysis.

### 2.7 Statistical analysis

Data were analyzed using SPSS (Version 25.0, SPSS Inc., Chicago, IL). Statistical differences among different treatments were determined by one-way ANOVA, and differences among different treatments were determined by Duncan’s multiple range test. The Tukey’s HSD test was used to analyze the *α* and *β* diversity of the rumen microbiota. The variance in labeled means without a common letter is significantly different (*p* ≤ 0.05).

## 3 Results

### 3.1 Impact of dietary supplementation with different sources of rumen RPM on goat growth performance

**Figure 1:**
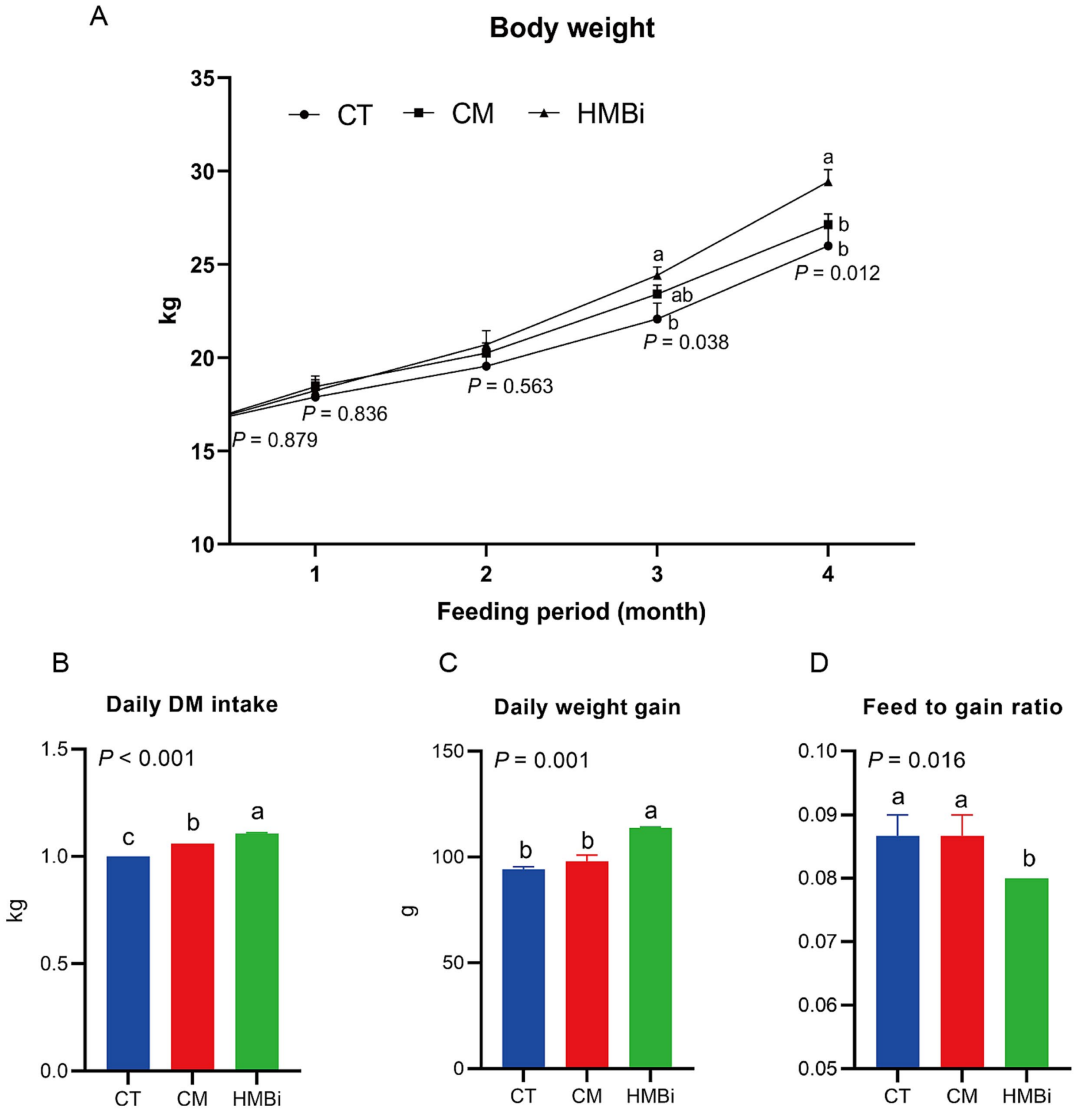
Effect of diet supplemented with different sources of RPM on growth performance. **(A)** Body weight changed with long-term feeding. **(B)** Daily DM intake. **(C)** daily weight gain. **(D)** Feed conversion ratio calculated by feed intake divided by weight gain. Labeled means without a common letter are significantly different, *p* ≤ 0.05. CT, goats fed basal diet; CM, goats fed coated methionine; HMBi, goats fed 2-hydroxy-4-(methylthio)-butanoic acid isopropyl ester.

In this study, HMBi supplementation significantly increased the goats body weight after 3 and 4 months of feeding when compared to the CT (*p* < 0.05, Figure 1A). At 3 and 4 months of HMBi feeding, the body weight increased by 10.55 and 13.19%, respectively. Though the CM supplementation showed an increase in numbers of body weight, no significant difference was found when compared to the CT (*p* > 0.05). Besides, we observed that a diet supplemented with HMBi significantly increased the daily DM intake (*p* < 0.05, Figure 1B) and daily weight gain (*p* < 0.05, Figure 1C), whereas CM supplementation significantly increased the daily DM intake (*p* < 0.05, Figure 1B). Furthermore, the administration of HMBi supplementation resulted in the feed-to-gain ratio (*p* < 0.05, Figure 1D).

### 3.2 Effects of dietary supplementation with different sources of RPM on the serum biochemistry of goats

The serum biochemistry was listed in Table 2. Results showed that diet supplemented with either CM or HMBi significantly increased the content of serum TC, BUN, ALP, and AST (all *p* < 0.05) and decreased the content of TG and BHB (all *p* < 0.05). No significant difference was found for the content of serum TP, ALB, GLB, and ALT and LDH (all *p* > 0.05).

**Table 2:** Effects of dietary supplements with different sources of RPM on serum metabolites^1^.

Items	CT	CM	HMBi	SEM	*P*-value
TC (mmol/L)	2.07^b^	2.34^a^	2.30^a^	0.047	0.035
TG (mmol/L)	0.31^a^	0.24^b^	0.19^b^	0.013	< 0.001
ALP (U/L)	523.78^b^	691.18^a^	719.84^a^	24.927	0.001
ALT (U/L)	12.64	12.06	12.78	0.424	0.448
AST (U/L)	98.98	110.18	104.85	1.941	0.057
LDH (U/L)	311.39^b^	321.80^ab^	344.07^a^	5.248	0.028
ALB (g/L)	27.67	26.93	27.79	0.237	0.284
TP (g/L)	70.47	69.87	71.49	0.536	0.475
BHB (mmol/L)	0.22^a^	0.18^b^	0.19^b^	0.033	0.043
GLB (g/L)	42.80	42.94	43.70	0.553	0.786
BUN (mmol/L)	2.63^b^	2.94^a^	3.14^a^	0.056	< 0.001

^1^CT, goats fed basal diet; CM, goats fed coated methionine; HMBi, goats fed 2-hydroxy-4-(methylthio)-butanoic acid isopropyl ester; TC, total cholesterol; TG, triglycerides; BUN, blood urea nitrogen; BHB, β-hydroxybutyric acid; TP, total protein; ALB albumin; GLB, globulin; ALP, alkaline phosphatase; ALT, alanine transaminase; AST, aspartate aminotransferase; LDH, lactic dehydrogenase. Labeled means without a common letter are significantly different, *p* ≤ 0.05.

### 3.3 Impact of dietary supplementation with different sources of RPM on rumen fermentation parameters

The rumen fermentation parameter was shown in Table 3. We found that diet supplemented with both CM and HMBi significantly increased the pH of the rumen fluid (*p* = 0.011). Besides, both CM and HMBi supplementation reduced the concentration of butyric acid (*p* = 0.007). However, both sources of methionine additives did not influence the concentration of acetic acid, propionic acid, acetic acid/propionic acid, isobutyric acid, isovaleric acid, pentanoic acid, or total VFA (all *p* > 0.05).

**Table 3:** Fermentation parameter determination^1^.

Items	CT	CM	HMBi	SEM	*P*-value
pH	7.29^b^	7.41^a^	7.42^a^	0.021	0.011
Acetic acid (mM)	7.81	7.52	7.71	0.217	0.861
Propionic acid (mM)	1.98	1.73	1.94	0.087	0.473
Acetic acid/Propionic acid	4.02	3.96	4.21	0.093	0.560
Isobutyric acid (mM)	0.90	0.84	0.91	0.025	0.458
Butyric acid (mM)	2.12^a^	1.53^b^	1.52^b^	0.094	0.007
Isovaleric acid (mM)	1.71	1.69	1.73	0.054	0.963
Pentanoic acid (mM)	2.56	2.63	2.52	0.041	0.520
Total VFA (mM)	17.08	15.94	16.31	0.405	0.518

^1^CT, goats fed basal diet; CM, goats fed coated methionine; HMBi, goats fed 2-hydroxy-4-(methylthio)-butanoic acid isopropyl ester. Labeled means without a common letter are significantly different, *P* ≤ 0.05.

### 3.4 Impact of dietary supplementation with different sources of RPM on rumen bacterial community richness and diversity

**Figure 2:**
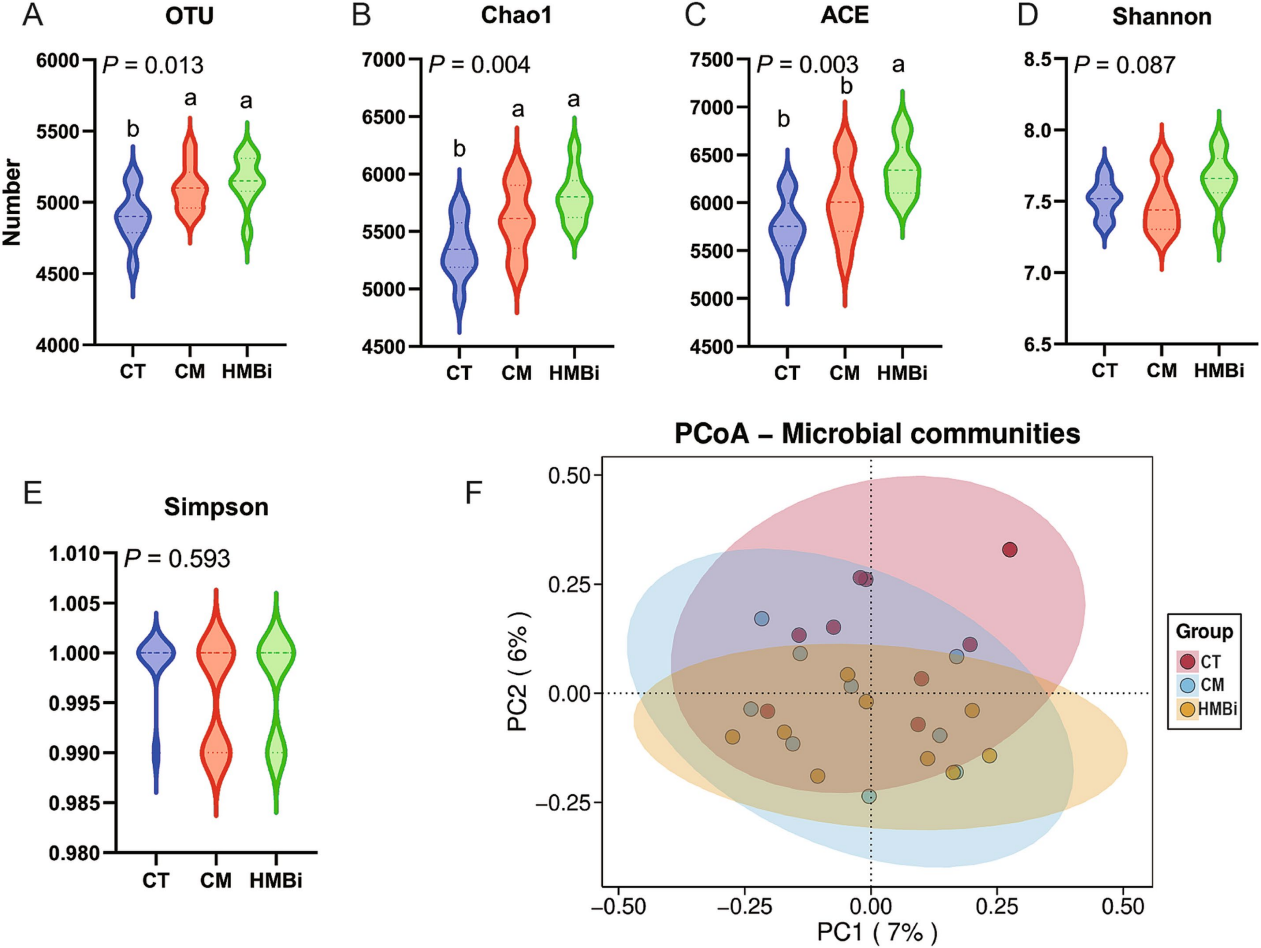
Effect of diet supplemented with different sources of RPM on *α* **(A–E)** and *β* diversity **(F)** of rumen microbes. CT, goats fed basal diet; CM, goats fed coated methionine; HMBi, goats fed 2-hydroxy-4-(methylthio)-butanoic acid isopropyl ester.

After merging and filtering, the 16S rRNA gene sequence analysis of the rumen content samples of goat in different treatments (30 samples) generated a total of 128,664 high-quality sequences, and OTUs were classified into 31 phyla, 67 classes, 129 orders, 252 families, and 678 genera. The *α*-diversity, including community richness indexes (OUT, Chao1, and ACE) and community diversity indexes (Shannon and Simpson), were shown in Figure 2. Results indicate that both CM and HMBi supplementation led to an increase in the levels of OUT and Chao1 (*p* < 0.05, Figures 2A,B). However, an increase in the ACE was only observed in HMBi supplementation (*p* < 0.01, Figure 2C). No difference was observed between Shannon and Simpson (*p* > 0.05, Figures 2D,E). The PCoA of the OTUs based on weighted UniFrac metrics revealed a distinct difference among the three groups (Figure 2F).

### 3.5 Impact of dietary supplementation with different sources of RPM on the composition of microbial communities and function prediction

**Figure 3:**
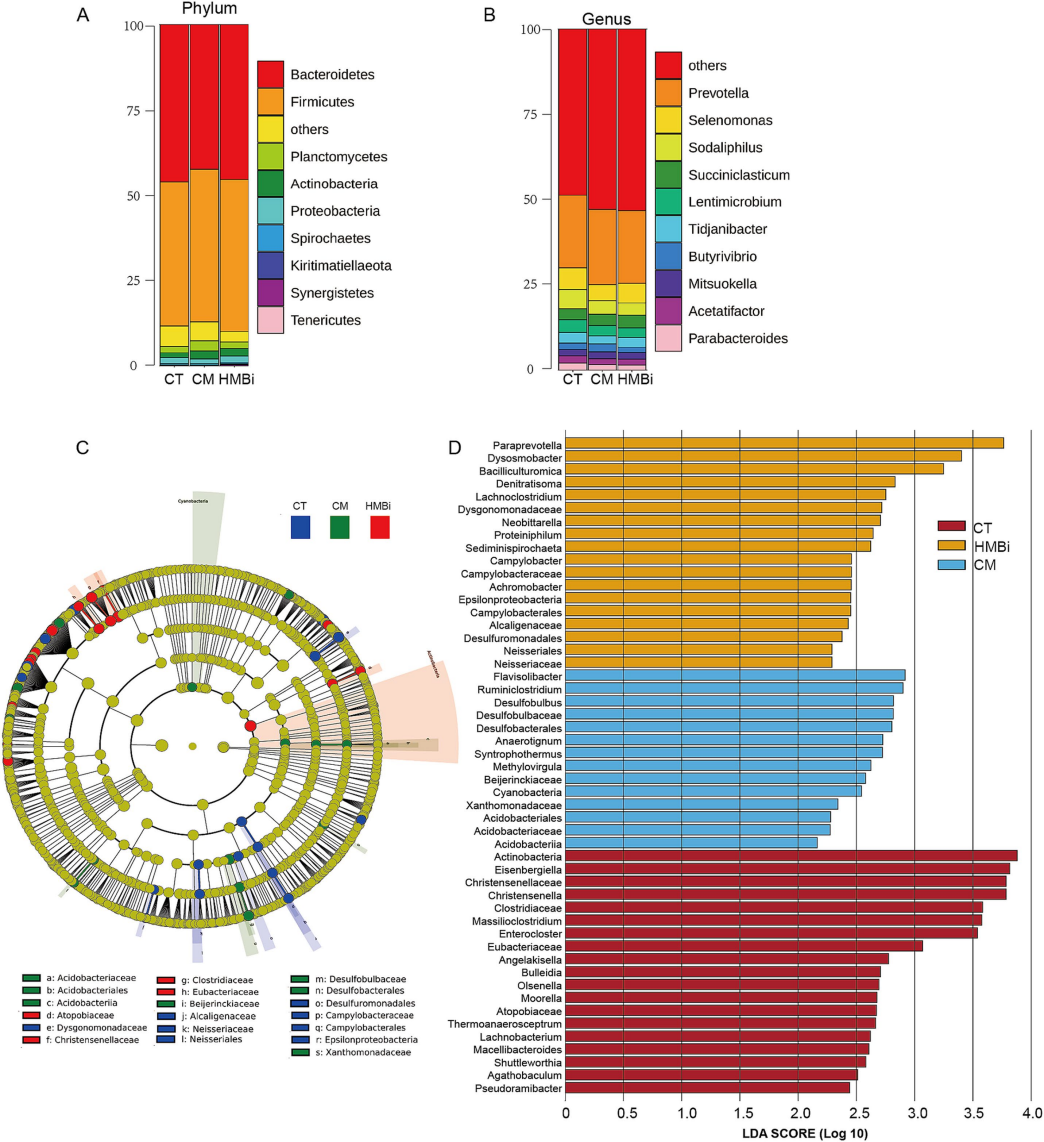
Effects of RPM treatment on the rumen bacterial community at the phylum and genus levels in weaned goats. **(A)** Alluvial chart at phylum and **(B)** genus level. **(C)** Cladogram of three groups. A cladogram showing the relationships among taxa at phylum, class, order, family, and genus levels was generated according to LEfSe analysis. **(D)** LDA score. The LDA score was derived from the LEfSe analysis, which showed that the biomarker taxa LDA score > 2 of rumen microbiota in three groups. CT, goats fed basal diet; CM, goats fed coated methionine; HMBi, goats fed 2-hydroxy-4-(methylthio)-butanoic acid isopropyl ester.

The top 10 most abundant bacteria at the phyla level of the ruminal fluid are presented in Figure 3A. We found that the dominant microbial phyla were *Bacteroidetes* (44.66%) and *Firmicutes* (43.76%), both of which ferment dietary fiber and produce VFA (19, 20). The top 10 genera with the highest relative abundance are shown in Figure 3B. The highest abundance genus was *Prevotella,* which accounts for 22.01%, followed by *Selenomonas* (4.73%), *Sodaliphilus* (3.86%), *Succiniclasticum* (3.28%), and *Lentimicrobium* (3.01%).

**Figure 4:**
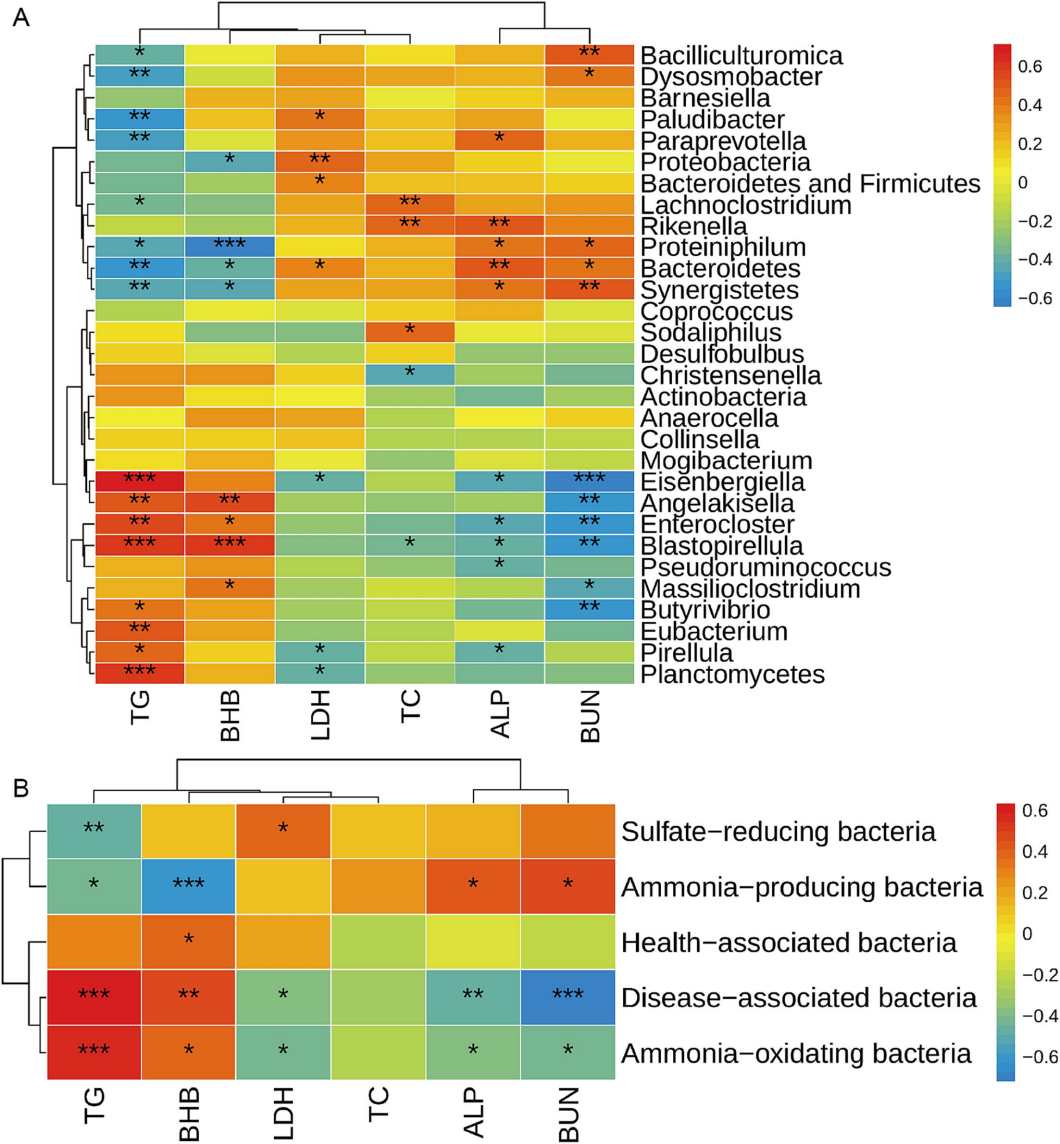
Correlation analysis between rumen bacteria and serum metabolites. The correlation between differential bacteria among the top 100 bacterial taxa and differential serum metabolites at genus level **(A)** and functional level **(B)** was analyzed by the Spearman method. Warm color represents a positive correlation, while cool color represents a negative correlation. *, **, and *** mean *p* ≤ 0.05, *p* ≤ 0.01, and *p* ≤ 0.001, respectively. TG, triglycerides; BHB, β-hydroxybutyric acid; LDH, lactic dehydrogenase; TC, total cholesterol; ALP, alkaline phosphatase; BUN, blood urea nitrogen.

In order to identify specific microbial biomarkers in the three groups, the data was analyzed using LEfSe selection with LDA scores above 2.0 and *p* < 0.05 among the three groups, and a cladogram was generated (Figures 3C,D) by LEfSe analysis of the rumen microbiota community. The most abundant bacterial taxa in the CT group were *Actinobacteria*, *Eisenbergiella*, *Christensenellaceae*, *Christensenella*, *Clostridiaceae*, *Massilioclostridium*, and *Enterocloster*. During CM treatment, the most differentially abundant bacterial taxa were *Flavisolibacter*, *Ruminiclostridium*, *Desulfobulbus*, *Desulfobulbaceae*, and *Desulfobacterales*. The most prevalent bacterial taxa in the HMBi treatment were *Paraprevotella*, *Dysosmobacter*, *Bacilliculturomica*, *Denitratisoma,* and *Lachnoclostridium*.

### 3.6 Impact of dietary supplementation with different sources of RPM on taxonomic differences of rumen microbiota

As shown in Table 4, it has been observed that both CM and HMBi resulted in a significant increase in the abundance of *Bacteroidetes* and *Proteobacteria* (both *p* < 0.05); however, only HMBi significantly increased the abundance of the sum of *Bacteroidetes* and *Firmicutes* (*p* < 0.05). Besides, both CM and HMBi supplementation resulted in a decrease in the abundance of *Planctomycetes* and *Actinobacteria* (*p* < 0.05). Furthermore, dietary HMBi increased the abundance of *Synergistete* (*p* < 0.05).

**Table 4:** Relative abundance (% of total reads) of rumen bacterial taxa at phylum in goats fed different sources of RPM^1^.

Taxon	CT	CM	HMBi	SEM	*P*-value
*Bacteroidetes*	42.44^b^	46.13^a^	45.42^a^	0.499	0.003
*Bacteroidetes + Firmcutes*	87.09^b^	88.31^ab^	89.64^a^	0.812	0.094
*Planctomycetes*	2.97^a^	1.77^b^	1.91^b^	0.956	0.005
*Actinobacteria*	2.36^a^	1.39^b^	1.73^b^	0.123	0.002
*Proteobacteria*	1.38^b^	1.84^a^	1.86^a^	0.090	0.040
*Synergistetes*	0.05^b^	0.07^b^	0.37^a^	0.04	< 0.001

^1^ The changed bacteria among the top 10 bacterial taxa were listed here. CT, goats fed basal diet; CM, goats fed coated methionine; HMBi, goats fed 2-hydroxy-4-(methylthio)-butanoic acid isopropyl ester. Labeled means without a common letter are significantly different, *P* ≤ 0.05.

**Figure 5:**
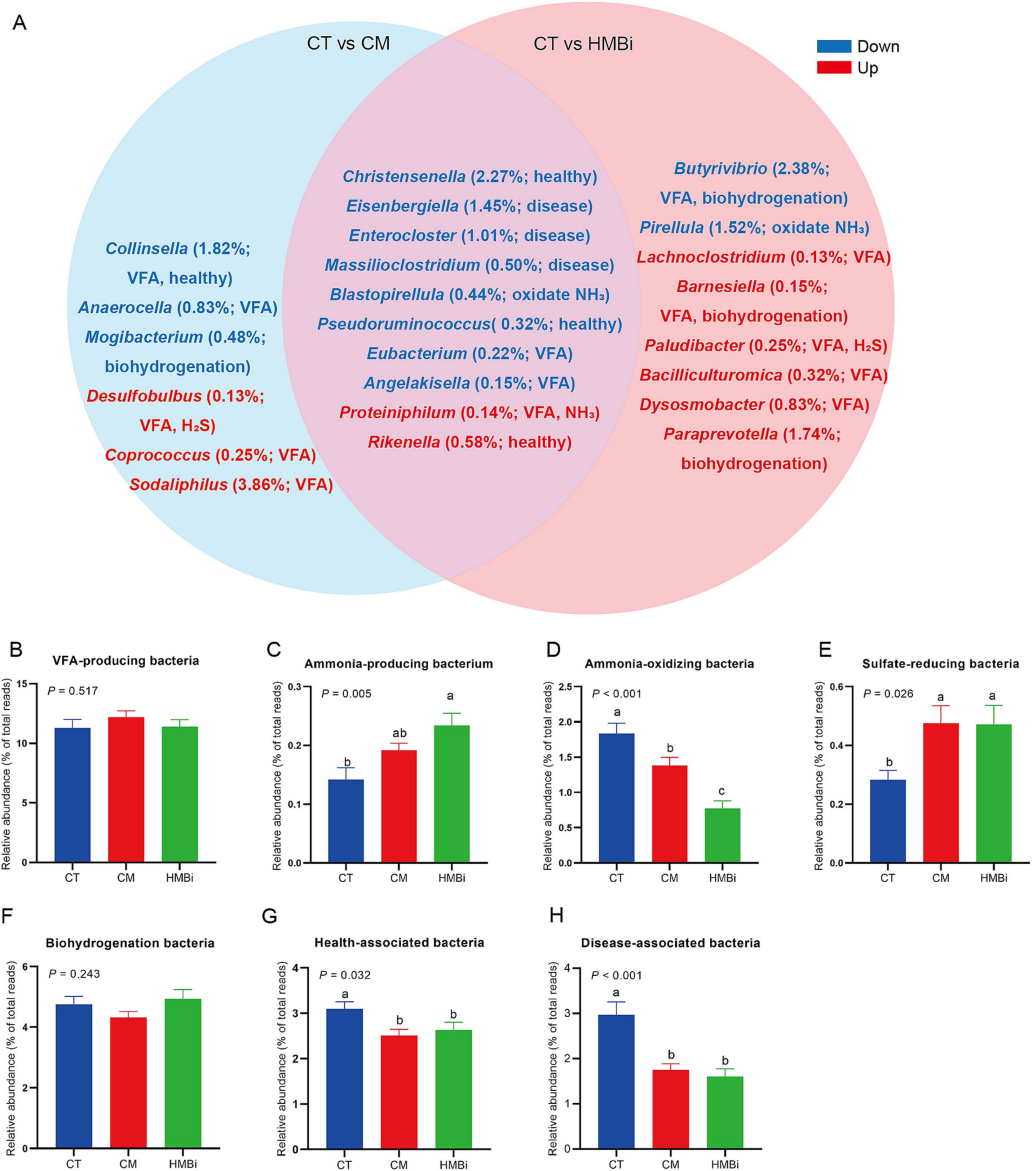
The differentially enriched taxon among the top 100 bacterial taxa among different treatments. **(A)** Venn diagram. Red font means higher abundance in treatments when compared to the CT, while blue font means lower abundance in treatments when compared to the CT. The relative abundance (% of total reads) of each bacterium and its function were listed within brackets. **(B–H)** Effect of diet supplemented with different sources of RPM on the abundance of **(B)** VFA-producing bacteria, **(C)** ammonia-producing bacteria, **(D)** ammonia-oxidating bacteria, **(E)** sulfate-reducing bacteria, **(F)** biohydrogenation bacteria, **(G)** health-associated bacteria, and **(H)** disease-associated bacteria. CT, goats fed basal diet; CM, goats fed coated methionine; HMBi, goats fed 2-hydroxy-4-(methylthio)-butanoic acid isopropyl ester; VFA, volatile fatty acid; H_2_S, hydrogen sulfide; NH_3_, ammonia.

We then examined the top 100 bacteria (relative abundance ≥0.05%) and found that the relative abundance of 24 bacteria was changed when goats were fed CM or HMBi (Table 5). As shown in the Venn diagram (Figure 5), it is found that both CM and HMBi supplementation increased the abundance of *Rikenella* and *Proteiniphilum* but decreased the abundance of *Eisenbergiella*, *Enterocloster*, *Massilioclostridium*, *Eubacterium* and *Angelakisella*, *Blastopirellula*, *Christensenella,* and *Pseudoruminococcus* (all *p* < 0.05). CM supplementation specially increased the abundance of *Desulfobulbus*, *Sodaliphilus,* and *Coprococcus* while decreasing the prevalence of *Anaerocella*, *Mogibacterium,* and *Collinsella* (all *p* < 0.05). HMBi supplementation significantly increased the abundance of *Paraprevotella*, *Bacilliculturomica*, *Lachnoclostridium*, *Dysosmobacter*, *Barnesiella*, and *Paludibacter* but decreased the abundance of *Butyrivibrio* and *Pirellula* (all *p* < 0.05).

**Table 5:** Relative abundance (% of total reads) of the differentially enriched rumen bacterial taxa at genus in goats fed different sources of RPM^1^.

Taxon	CT	CM	HMBi	SEM	*P*-value
*Butyrivibrio*	2.38^a^	1.92^ab^	1.57^b^	0.112	0.008
*Christensenella*	2.27^a^	1.43^b^	1.48^b^	0.584	< 0.001
*Eisenbergiella*	1.45^a^	0.98^b^	0.79^b^	0.080	0.001
*Enterocloster*	1.01^a^	0.52^b^	0.53^b^	0.059	< 0.001
*Massilioclostridium*	0.50^a^	0.25^b^	0.28^b^	0.044	0.041
*Mogibacterium*	0.48^a^	0.29^b^	0.43^ab^	0.032	0.047
*Pseudoruminococcus*	0.32^a^	0.2^b^	0.17^b^	0.025	0.029
*Bacilliculturomica*	0.32^b^	0.47^ab^	0.62^a^	0.044	0.014
*Dysosmobacter*	0.25^b^	0.38^b^	0.82^a^	0.057	< 0.001
*Coprococcus*	0.25^b^	0.40^a^	0.25^b^	0.028	0.039
*Eubacterium*	0.22^a^	0.08^b^	0.12^b^	0.020	0.005
*Angelakisella*	0.15^a^	0.06^b^	0.05^b^	0.014	0.005
*Lachnoclostridium*	0.13^b^	0.17^b^	0.24^a^	0.013	0.002
*Sodaliphilus*	3.86^b^	5.61^a^	3.40^b^	0.316	0.007
*Paraprevotella*	1.74^b^	1.95^b^	2.61^a^	0.113	0.017
*Anaerocella*	0.83^a^	0.40^b^	0.86^a^	0.081	0.029
*Rikenella*	0.58^b^	0.89^a^	0.99^a^	0.062	0.017
*Paludibacter*	0.17^b^	0.26^ab^	0.41^a^	0.041	0.051
*Barnesiella*	0.15^b^	0.15^b^	0.31^a^	0.02	< 0.001
*Proteiniphilum*	0.14^b^	0.19^a^	0.23^a^	0.012	0.005
*Collinsella*	1.82^a^	1.23^b^	1.66^a^	0.083	0.007
*Pirellula*	1.52^a^	1.30^a^	0.66^b^	0.176	0.026
*Blastopirellula*	0.44^a^	0.16^b^	0.14^b^	0.044	0.004
*Desulfobulbus*	0.13^b^	0.21^a^	0.08^b^	0.017	0.009

^1^ Differential taxon at genus among the top 100 bacterial taxa were listed. CT, goats fed basal diet; CM, goats fed coated methionine; HMBi, goats fed 2-hydroxy-4-(methylthio)-butanoic acid isopropyl ester. Labeled means without a common letter are significantly different, *P* ≤ 0.05.

According to the function of each different genera (Figure 5A), we found that both CM and HMBi increased the abundance of sulfate-reducing bacteria (*p* < 0.05, Figure 5E) and ammonia-producing bacteria (*p* < 0.05, Figure 5C), but decreased the abundance of ammonia-oxidizing bacteria (*p* < 0.05, Figure 5D), health-associated bacteria (*p* < 0.05, Figure 5G), and disease-associated bacteria (*p* < 0.05, Figure 5H). However, both CM and HMBi did not have an influence on the abundance of VFA-producing bacteria (*p* > 0.05, Figure 5B) and biohydrogenation bacteria (*p* > 0.05, Figure 5F).

### 3.7 Correlations between rumen bacteria and serum metabolites

Gut bacteria are complex, and the disorder of the bacterial community usually causes diseases such as neurodegenerative diseases, cardiovascular diseases, metabolic diseases, and gastrointestinal diseases (9, 10, 21). Blood metabolites are always used as an index to reflect the most common diseases. So, we further analyzed the correlations between rumen bacteria and serum metabolites. The correlation between the affected ruminal microbiota (at phylum and genus level) and serum metabolites was analyzed by Spearman’s correlation analysis (Figure 4A). Serum TG showed a positive correlation with *Planctomycetes*, *Eubacterium*, *Pirellula*, *Angelakisella*, *Butyrivibrio*, *Blastopirellula*, *Enterocloster,* and *Eisenbergiella*, while it was negatively correlated with *Bacteroidetes*, *Synergistetes*, *Proteiniphilum*, *Paludibacter*, *Lachnoclostridium*, *Paraprevotella*, *Bacilliculturomica,* and *Dysosmobacter*. BHB was positively correlated with *Massilioclostridium*, *Angelakisella*, *Blastopirellula*, and *Enterocloster*, while it was negatively correlated with *Bacteroidetes*, *Synergistetes*, *Proteobacteria,* and *Proteiniphilum*. BUN was positively correlated with *Bacteroidetes*, *Synergistetes*, *Proteiniphilum*, *Bacilliculturomica,* and *Dysosmobacter*, while it was negatively correlated with *Massilioclostridium*, *Angelakisella*, *Butyrivibrio*, *Blastopirellula*, *Enterocloster,* and *Eisenbergiella*. Serum TC was positively correlated with *Lachnoclostridium*, *Rikenella,* and *Sodaliphilus*, while it was negatively correlated with *Christensenella* and *Blastopirellula*. Serum LDH was positively correlated with *Proteobacteria*, the sum of *Bacterioidetes* and *Firmicutes*, *Bacterioidetes*, and *Plaudibacter*, while it was negatively correlated with *Planctomycetes*, *Pirellula,* and *Eisenbergiella*. Serum ALP was positively correlated with *Bacteroidetes*, *Synergistetes*, *Proteiniphilum*, *Rikenella*, and *Paraprevotella*, while it was negatively correlated with *Pseudoruminococcus*, *Pirellula*, *Blastopirellula*, *Enterocloster,* and *Eisenbergiella*.

Furthermore, the correlation of serum metabolites and functional bacteria was analyzed by Sperson analysis (Figure 4B). We observed that serum TG was positively correlated with disease-associated bacteria and ammonia-oxidating bacteria, and negatively correlated with sulfate-reducing bacteria and ammonia-producing bacteria. Serum BHB was positively correlated with disease-associated bacteria, health-associated bacteria and ammonia-oxidating bacteria, while negatively correlated with ammonia-producing bacteria. The serum ALP was negatively correlated with disease-associated bacteria and ammonia-oxidating bacteria, and positively correlated with ammonia-producing bacteria. BUN was negatively correlated with disease-associated bacteria, ammonia-oxidating bacteria and positively correlated with ammonia-producing bacteria.

### 3.8 Impact of dietary supplementation with different sources of RPM on the morphology of the small intestine

**Figure 6:**
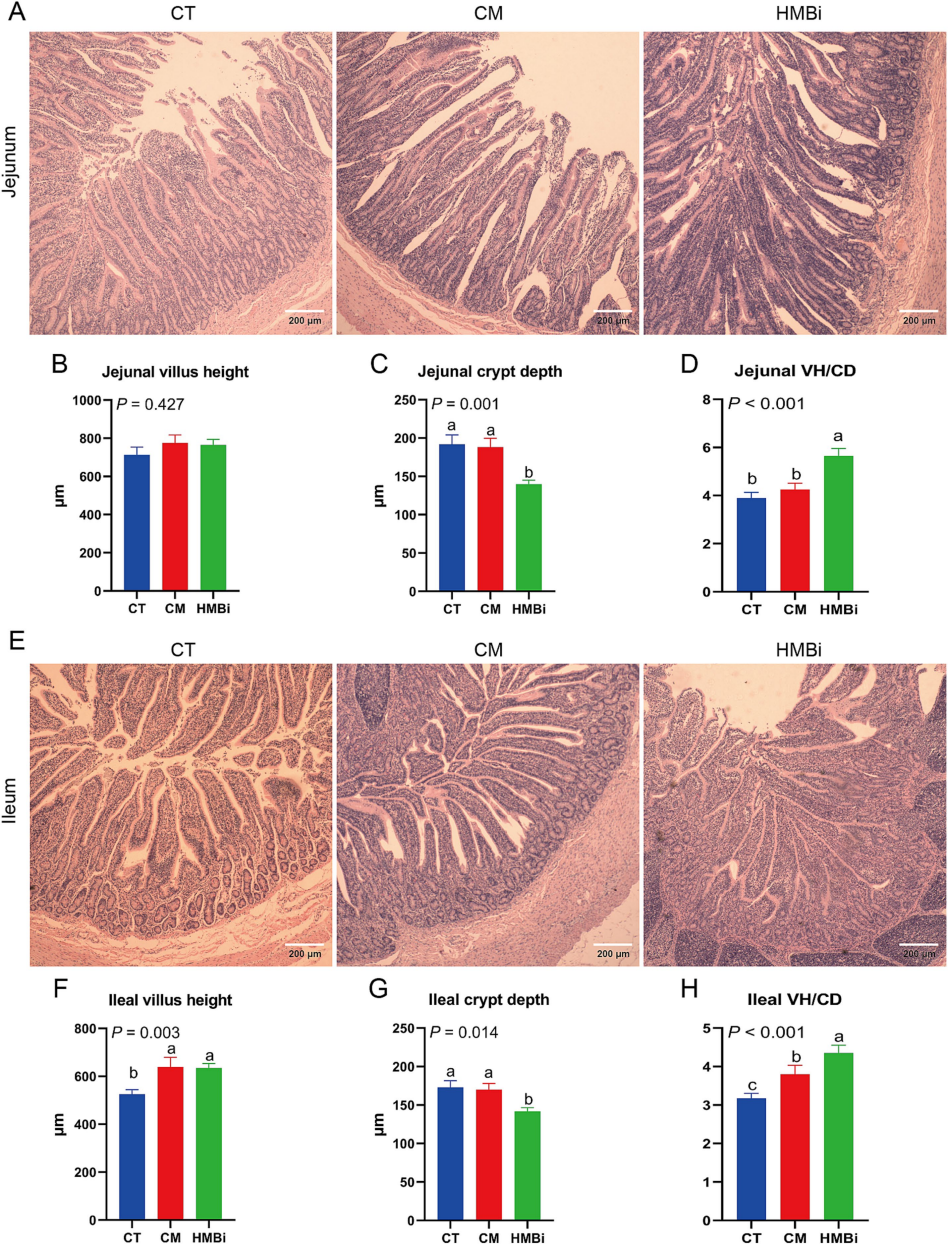
Morphology of the small intestine. HE staining was used to detect the villus height and crypt depth in the jejunum and ileum. **(A-D)** Villus height, crypt depth, and VH/CD of jejunum. **(E-H)** Villus height, crypt depth, and VH/CD of ileum. Different letters on bars mean a significant difference (*p* ≤ 0.05). VH/CD, villus height to crypt depth ratio; CT, goats fed basal diet; CM, goats fed coated methionine; HMBi, goats fed 2-hydroxy-4-(methylthio)-butanoic acid isopropyl ester.

Intestine is an important site for nutrient absorption, such as microproteins synthesized by the rumen microbes (22). So, we further investigated the influence of different sources of RPM on the development of the small intestine (Figure 6). The results showed that supplementation with HMBi decreased the CD (*p* < 0.05) and increased the VH to CD ratio (VH/CD, *p* < 0.05) in jejunums when compared to the CT. In ileum, we observed that both CM and HMBi supplementation increased the VH and VH/CD (both *p* < 0.05), but only HMBi supplementation significantly decreased the CD (*p* < 0.05).

## 4 Discussion

RPM is widely used in dairy ruminant animals, such as dairy cows and dairy goats. Studies have shown that diet supplementation with RPM improves milk fat and protein (5). However, the effect of RPM on meat goats is unclear. Rumen contains a great diversity of microbiota and is a major site for digestion of diet cellulose and hemicelluloses. It also produces VFA and microbial proteins in ruminant. VFA is mainly absorbed by the rumen, and microbial proteins are usually absorbed by the abomasum and intestine. Therefore, this study found that both CM and HMBi improved serum lipid metabolism, the composition of the rumen microbe community, and intestinal development, but HMBi performed better than CM. This may explain the improved growth performance in HMBi.

The body weight is the key index for efficiency assessment. Recent studies conducted on growing goats have demonstrated that CM supplementation at a concentration between 0.1 and 1.15% has no impact on body weight (8–15). In this study, diet supplemented with CM at 0.12% had no effect on body weight, even after a longer feeding period was observed. It appears that CM supplementation has no influence on body weight, even if it is fed for a long time. No weight gain has been reported in goats eating HMBi (8). In this study, feeding periods within 2 months showed no difference between goats fed HMBi and basal diets. However, a significant difference was observed after 3 months of feeding period, and the growth rate gradually increased with an extended feeding period. Besides, we found that daily weight gain, daily DM intake, and ratio of feed to weight gain were improved in goats fed HMBi as well, which is similar to the report in goats (8) and Holstein calves (23). Dietary methionine restriction has been proven to extend lifespan, and a high level of methionine intake shows adverse effects such as hyperhomocysteinemia, reduced body weight, and increased cholesterol levels (24). However, in this study, a diet supplemented with CM or HMBi did not reduce body weight but increased the level of serum TC. So, considering that no negative effect was observed in body weight when goats were fed CM or HMBi, we speculated that the dosage of RPM in this study was bearable for goats. In summary, these results indicate that 3-months of feeding of HMBi at 0.22% is necessary for improved growth performance and that CM supplementation has no influence on growth performance.

Serum metabolites usually reflect the body’s health. Usually, TC and TG, the main serum lipids, are regarded as causes of cardiovascular disease (25, 26). In this study, both CM and HMBi supplementation decreased the serum TG, which is similar to results from adult ewes fed HMBi (16) and lactating dairy cows (27). This implies that both CM and HMBi supplementation improve the serum lipid metabolism. ALP, ALT, AST, and LDH are serum indices of liver function (28). ALT and AST mainly exist in parenchymal liver cells, with a low level in the blood, while ALP and LDH were increased in impaired bile excretion and hepatitis, respectively. In this study, both CM and HMBi supplementation increased the serum ALP and LDH, which is similar to the studies in yaks (29) and rats (30). Besides, serum ALP is positively correlated with the growth-promoting effect in yaks fed HMBi (29). Similarly, an increase in body weight was observed in goats fed HMBi. So, the improved growth performance may be at the expense of liver damage. The BHB is an indicator of ketonemia. Higher BHB is usually caused by glycopenia, hyperlipidemia, and proteolysis during negative energy balance (31). In this study, we found that both CM and HMBi supplementation decreased the serum BHB, which suggests that the anti-ketonemia ability is strengthened during CM or HMBi supplementation. Besides, it was observed that both CM and HMBi increased BUN, which is similar to the findings reported in cattle (6). BUN reflects the rumen ammonia production. In the rumen, the so-called ‘anammox’ *Planctomycetes* have a unique ability to oxidize ammonium to nitrogen (32), and *Proteiniphilum* utilize nitrogen to synthesize ammonia (33). In this study, both CM and HMBi supplementation decreased the abundance of *Planctomycetes* but increased the abundance of *Proteiniphilum*, which means more ammonia was produced. Besides, a report has shown that both CM and HMBi supplementation increase nitrogen utilization (34). So, the increased BUN during RPM supplementation implies improved nitrogen utilization in the rumen. In summary, CM and HMBi supplementation improve the serum lipid metabolism and nitrogen utilization and decrease the risk of ketonemia, but increase the risk of liver damage.

VFA produced by rumen microbes is mainly containing acetic acid, propionic acid, isobutyric acid, butyric acid, isovaleric acid, and pentanoic acid (35). The VFA was absorbed mainly by rumen, and the rest was absorbed by omasum and abomasum. So, the concentration of rumen VFA is determined by microbial fermentation and the absorption of rumen, omasum, and abomasum. Dietary CM and HMBi decreased concentrations of butyric acid were found in both CM and HMBi supplementation in this study, which is similar to the study conducted on mice (36). Inversely, study in cows has shown that CM feeding increased ruminal butyrate concentration (11, 12). The decreased concentration of butyric acid may result from the decreased abundance of *Butyrivibrio*, a key bacterium for butyric acid production (37). Even though increased feed intake and fiber-degrading bacteria (sum of *Bacteroidetes* and *Firmicutes*) were observed in goat-fed HMBi in this study, no difference was found in the concentration of rumen VFA. Similarly, no difference was also found in lactating cows (38, 39). A recent study has shown that methionine benefits the growth of epithelial cells in the rumen and intestine (4). Here, we think that HMBi supplementation increases the VFA-producing bacteria, accompanied by an increased absorption rate of VFA in rumen.

A highly diverse rumen microbiota is generally considered beneficial for host health and is regarded as a sign of a mature rumen microbiota (40). In this study, alpha diversity indices (OUT and Chao1) of the rumen microbiota significantly increased in goats fed CM or HMBi. Besides, it’s reported that high pH is accompanied by high bacterial diversity (41). Similarly, increased rumen pH was observed in goats fed CM or HMBi in this study.

At phylum level, *Bacteroidetes* (44.66%) and *Firmicutes* (43.76%) were the dominant phyla in rumen and accounted for the majority of the microbial community in Jianzhou Da’er goats, which is similar to Hu sheep, Tan sheep, Dorper sheep, and back Bengal goats (42, 43). *Bacteroidetes* and *Firmicutes* are renowned for their ability to degrade a wide range of complex carbohydrates to produce VFA (19, 20). In this study, goats fed HMBi showed a higher level of the sum of *Bacteroidetes* and *Firmicutes*, which means HMBi benefits for carbohydrates broken down and VFA production. This explained the high daily feed intake in the HMBi group. *Planctomycetes* can oxidate ammonia into nitrogen (32) and *Proteobacteria* is a nitrogen-fixing bacterium (44). In this study, both HMBi and CM supplementation decreased the abundance of ammonia-oxidating bacterium *Planctomycetes* and increased the nitrogen-fixing bacterium *Proteobacteria*, which means that both HMBi and CM supplementation benefit for a high level of rumen ammonia. This explained the high BUN in goat-fed CM and HMBi. Various genera in *Actinobacteria* (45) and *Proteobacteria* (46) have the ability to fight pathogenic bacteria. In this study, the increased pathogen-against bacterium *Proteobacteria* offset the decreased abundance of *Actinobacteria*. *Synergistetes* can use amino acids to produce VFA (47) and also have genes for lipopolysaccharide biosynthesis (48). In summary, at the phylum level, these findings suggest that both CM and HMBi supplementation have a positive impact on ammonia production, with HMBi showing a particular benefit in the breakdown of carbohydrates.

Most rumen bacteria in *Firmicutes* and *Bacteroidetes* can hydrolyze dietary fiber and produce VFA (19, 20). In this study, even though most of the differential bacteria were VFA-producing bacteria, such as *Butyrivibrio* (49), *Bacilliculturomica* (50), *Dysosmobacter* (51), *Coprococcus* (14), *Eubacterium* (52), *Angelakisella* (53), *Lachnoclostridium* (54, 55), *Sodaliphilus* (56), *Anaerocella* (57), *Barnesiella* (58) and *Collinsella* (17, 18), the sum of these bacteria showed that both CM and HMBi did not influence the abundance of the VFA-producing bacteria, which explains the result of total VFA in rumen fluid. Besides, ruminant diet mainly contains 18-carbon unsaturated fatty acids, such as *α*-linolenic acid, linoleic acid, and oleic acid. Under the biohydrogenation of bacteria such as *Paraprevotella* (59) *Barnesiella* (58), and *Mogibacterium* (60), unsaturated fatty acid was converted to saturated fatty acid, followed by hydrogenation of the double bonds (61). Even though a low biohydrogenation is warranted for a product rich in unsaturated fatty acids (62), no difference was found during CM and HMBi supplementation. So, CM and HMBi supplementation showed no influence on rumen biohydrogenation. Besides, rumen bacteria ferment nitrogenous substances into ammonia, and the ammonia was then used for the synthesis of microbial protein by ammonia-producing bacteria and then flowed into the hindgut to be absorbed (22). Besides, a part of ammonia was oxidated into nitrogen by ammonia-oxidating bacteria (33). In this study, the ammonia-producing bacteria *Proteiniphilum* (33) was increased when diet supplemented with HMBi. Besides, both CM and HMBi decreased the ammonia-oxidating bacteria, including *Pirellula* (63) and *Blastopirellula* (64). Similarly, studies in cows have shown that HMBi supplementation increases nitrogen efficiency (34) and milk protein (5). These results imply that CM and HMBi have a positive impact on nitrogen utilization, which is similar with the results of analysis at phyla and explained the increased BUN as well. Dietary sulfur can be reduced into hydrogen sulfide (H_2_S) in rumen by sulfate-reducing bacteria, such as *Desulfobulbus* (65) and *Paludibacter* (66). H_2_S is a toxic gas that can be quickly absorbed by the rumen wall, and the accumulation of excessive H_2_S in the rumen may pose toxicity risks to ruminant animals (67). It has been shown that the level of dietary sulfur was positively correlated with H_2_S production (68). Methionine is a sulfur-containing amino acid. Furthermore, our study revealed distinct mechanisms by which CM and HMBi enhance H_2_S production, with CM promoting an increase of *Desulfobulbus* and HMBi facilitating an increase of *Paludibacter*. In this study, it was observed that both CM and HMBi led to an increase in sulfate-reducing bacteria, which means increased H_2_S production. What’s more, we found that both CM and HMBi supplementation decreased the abundance of disease-associated bacteria *Eisenbergiella* (69), *Enterocloster* (70) and *Massilioclostridium* (71), and the health-associated bacteria *Christensenella* (72), *Pseudoruminococcus* (54, 55) and *Rikenella* (73–75). The decreased health-associated bacteria may be a responsive effect of decreased disease-associated bacteria. So, CM and HMBi supplementation benefit body health. In summary, the supplementation of RPM has been found to have positive effects on ammonia and H_2_S production and decreased the disease risk.

The small intestine plays an important role in absorbing microbial protein that is produced in rumen (3). Generally, higher VH means greater absorption area, while shallow CD means fast villus growth rate (76). A study in sows showed that methionine or HMBi supplementation increased the VH and the ratio of VH/CD of the piglets (77). In this study, we found that both CM and HMBi increased ileal VH and HMBi supplementation decreased CD, with HMBi doing better. Previous work has shown that methionine benefits isolated crypt development (4), so the improved intestine development here may be caused by the methionine-induced crypt development. In summary, these results imply that both CM and HMBi supplementation improve intestinal morphology, especially HMBi supplementation.

## 5 Conclusion

This study indicates that both CM and HMBi supplementation improve lipid metabolism, nitrogen utilization, and intestinal development. In contrast to CM supplementation, the growth-promoting effect of HMBi supplementation may be attributed to the greater abundance of rumen VFA-producing bacteria, nitrogen-utilizing bacteria, and enhanced intestinal development. This study suggests that HMBi is a good methionine additive for meat goats.

## Data Availability

The original contributions presented in the study are included in the article/supplementary material, further inquiries can be directed to the corresponding author.
